# Supplementary material for: Don’t Forget the Bones: Incidence and Risk Factors of Metabolic Bone Disease in a Cohort of Preterm Infants
Source: Int J Mol Sci. 2022 Sep 14;23(18):10666. doi: 10.3390/ijms231810666 (PMC9506409; doi:10.3390/ijms231810666)
Supplement: Supplementary file 1 [file ijms-23-10666-s001.zip › ijms-1873095-supplementary.pdf]

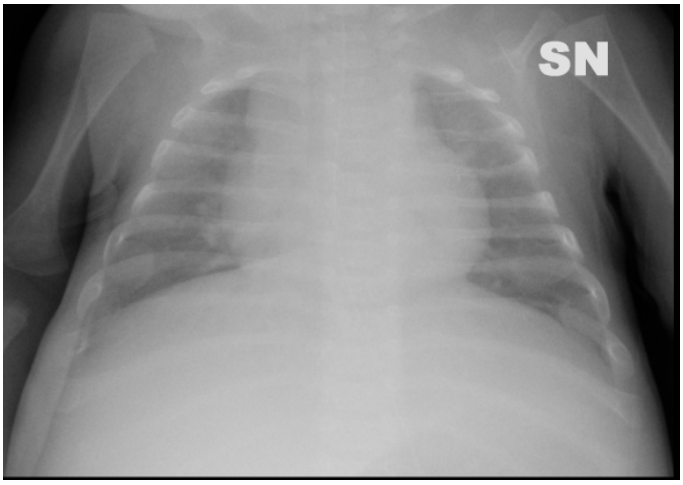

Figure S1 - Patient a, GA 29+3, BW 650 g, male.

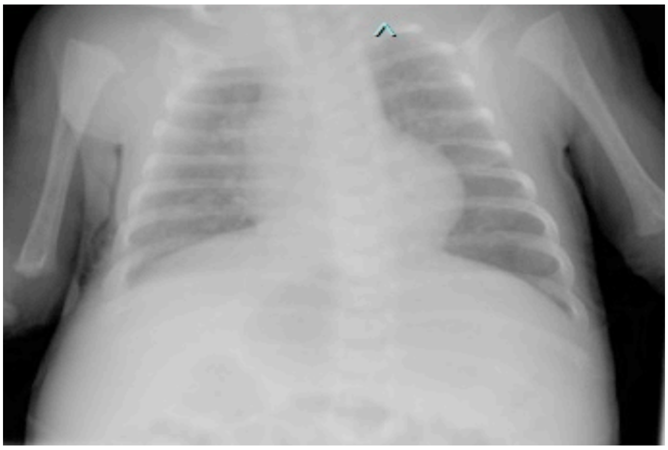

Figure S2 - Patient b, GA 28+6, BW 570 g, female.

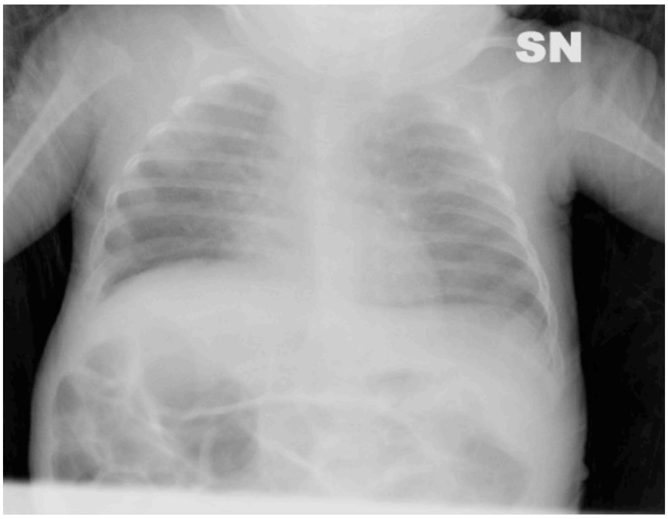

Figure S3 - Patient c, GA 28+3, BW 640 g, male.

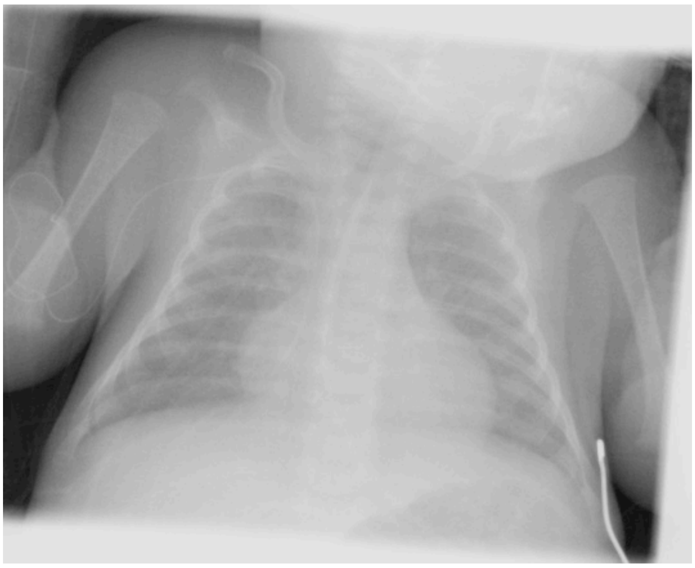

Figure S4 - Patient d, GA 30+3, BW 960 g, female.

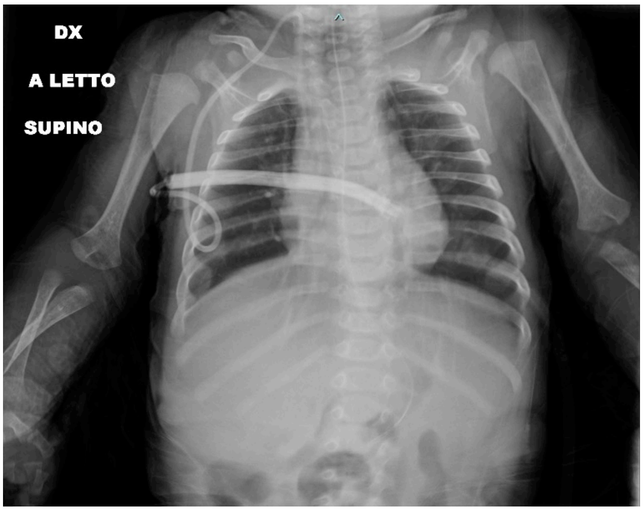

Figure S5 - Patient e, GA 29+1, BW 890 g, male.
